# Commentary: The Role of the Parietal Cortex in the Representation of Task–Reward Associations

**DOI:** 10.3389/fnhum.2016.00192

**Published:** 2016-05-10

**Authors:** Elger L. Abrahamse, Massimo Silvetti

**Affiliations:** ^1^Department of Experimental Psychology, University of GhentGhent, Belgium; ^2^Ghent Institute for Functional and Metabolic Imaging, Ghent University HospitalGhent, Belgium

**Keywords:** inferior parietal cortex (IPC), reward, temporal parietal junction, state prediction, task-reward association

Broadly linked to domains such as attention (Corbetta and Shulman, 2002), episodic memory (Cabeza et al., 2008) and language (Price, 2010), profiling of the heterogeneous inferior parietal cortex (IPC) is challenging. Here we comment on a recent IPC paper by Wisniewski et al. (2015). Briefly, we believe that their main findings may be parsimoniously explained by existent theories on IPC, whereas another aspect of their study deserves more attention.

Wisniewski and colleagues explored the brain areas underlying associations between tasks (parity/magnitude) and rewards (high/low). The information required for task execution gradually unfolded across a trial: Participants subsequently received a symbolic *task-reward association cue* (2-s) followed by a 6-s delay (phase 1), a fully informative *task cue* (1.5-s) followed by a 1.5-s delay (phase 2), and the *target stimulus* followed by some time to respond (phase 3)^1^. Finally, successful performance was rewarded accordingly. Two major observations resulted from fMRI-based multi-voxel pattern analysis. First, during phase 1 the bilateral IPC coded for the instructed task-reward associations. Second, IPC coding was adaptive as IPC later in the trial also coded for reward prospect (~phase 2/3) and (in left IPC) task-to-be-performed (~phase 3). This exposes IPC as a critical link between cognitive control and motivational functions in the brain. Yet, below we critically discuss how these findings contribute to our knowledge about IPC.

Phase 1 required the symbolically cued task-reward associations to be maintained for 6-s until task cue presentation. Robust coding was observed in bilateral IPC, covering parts of both the supramarginal gyrus (SMG) and the angular gyrus (AG). The current literature links these areas to semantic and phonological processing; for example, left SMG supports verbal working memory (Deschamps et al., 2014) including phonological maintenance (Sliwinska et al., 2012), and AG underlies semantic processing and the construction of meaning (Seghier, 2013). Indeed, right IPC is also often observed in working memory tasks (Owen et al., 2005)—possibly linked to spatial rehearsal (Smith and Jonides, 1998). With a symbolic task-reward association cue requiring maintenance (either in visuo-spatial or verbal format) and (at some point) semantic translation, the bilateral IPC coding during phase 1 may be directly linked to well-known roles in the context of a (verbal) working memory task.

Although interesting, the adaptation of IPC coding from phase 1 to 3 may not be surprising from the perspective taken above. If left IPC, for example, is involved in the processing and maintenance of verbal content, then its content may switch within trials because the currently relevant information is updated. A similar argument can be made for additional types of format (e.g., visuo-spatial information). More generally, with the heterogeneity of inferior parietal cortex in mind, what does it mean to conclude that “the inferior parietal cortex flexibly changed its content of representation on a short timescale within trials” (p. 12363; Wisniewski et al., 2015)? These are issues to consider. Moreover, we believe that a particularly interesting feat of phase 2/3 was not elaborated on by Wisniewski and colleagues.

A ventral fronto-parietal circuit involving temporal parietal junction (TPJ) and the inferior frontal gyrus (IFG)—initially linked to attentional reorientation *per se*—has recently been speculated to *evaluate* (the matching or mismatching of) attentional templates (Doricchi et al., 2010; Han and Marois, 2014). The study by Wisniewski and colleagues fits well with this framework. Specifically, maintaining information in memory enables the formulation of predictions about upcoming states of the overall task context. We can hypothesize, then, that after the first cue subjects formulated a set of predictions about future task and reward states such that the second cue became an outcome triggering a comparison with the previously formulated predictions. Prediction-outcome comparison for high vs. low reward can explain the bilateral involvement of the TPJ (and IFG) observed across phase 2/3.

And there is more. Using endogenous Posner tasks, Doricchi and colleagues (Doricchi et al., 2010; Silvetti et al., 2015) observed that in the left TPJ anatomically overlapping though functionally separated neuronal populations coded for valid (matching attentional expectation) and invalid (mismatching attentional expectation) targets, whereas the right TPJ seemed predominantly sensitive to invalid targets. Interestingly, Wisniewski and colleagues—using cues of a different type and a different task context—more or less replicated this intriguing left-right TPJ difference. Specifically, the task coding observed in their study for phase 3 in the left but not right TPJ may be directly linked to the fact that with a fully predictive task cue the target stimulus never generated broken predictions (in strong analogy to valid Posner trials). This suggests—besides the general notion that TPJ compares predictions on states of the environment with actual outcomes—that left and right TPJ perhaps have equally broad but (partly) different roles. The large overlap in coding between highly diverse events as Posner cues (Silvetti et al., 2015) and task (and reward) state cues (Wisniewski et al., 2015) strongly probes our curiosity about what exactly are the respective roles of left and right TPJ. This issue may benefit from future exploration of TPJ coding with an increasingly broader lens, crossing broadly related domains as mentalizing (Frith and Frith, 2006), social evaluation (Decety and Lamm, 2007), oddball processing (Cabeza et al., 2012), and integration of top-down and bottom-up control (Wu et al., 2015). Is prediction-outcome comparison the common denominator of TPJ involvement across these domains? It may be. But pinpointing left-right TPJ differences will possibly turn out to be an even bigger challenge.

In sum, our comment is twofold. First, findings by Wisniewski and colleagues fit well with existent knowledge on parietal cortex, thereby questioning the aspect of novelty. Second, and more importantly, their study further prompts comprehensive exploration of TPJ in terms of prediction-outcome comparison—surpassing the level of specific domains, and considering the potentially distinct contributions of left and right TPJ.

## Author contributions

All authors listed, have made substantial, direct and intellectual contribution to the work, and approved it for publication.

### Conflict of interest statement

The authors declare that the research was conducted in the absence of any commercial or financial relationships that could be construed as a potential conflict of interest. The reviewer GA and handling Editor declared their shared affiliation, and the handling Editor states that the process nevertheless met the standards of a fair and objective review.

## Footnotes

^1^Unlike the original study, we divide trials into three phases. This corresponds to the data, although exact timing of effects is impossible due to unknown hemodynamic lag.
